# *Aeromonas caviae* mimicking *Vibrio cholerae* infectious enteropathy in a cholera-endemic region with possible public health consequences: two case reports

**DOI:** 10.1186/s13256-018-1603-5

**Published:** 2018-03-17

**Authors:** Marco van Zwetselaar, Balthazar Nyombi, Tolbert Sonda, Happiness Kumburu, Nyasatu Chamba, Marieke C. J. Dekker, Kajiru G. Kilonzo, Sarah J. Urasa, Blandina T. Mmbaga

**Affiliations:** 1 Kilimanjaro Clinical Research Institute, Moshi, Kilimanjaro United Republic of Tanzania; 2 Kilimanjaro Christian Medical Centre, Moshi, Kilimanjaro United Republic of Tanzania; 3 Kilimanjaro Christian Medical University College, Moshi, United Republic of Tanzania

**Keywords:** Diarrhea, Cholera outbreak, *Aeromonas spp.*, *V. cholerae*, Whole genome sequencing

## Abstract

**Background:**

*Aeromonas* species have been documented to yield false positive results in microbiological tests for *Vibrio cholerae*. They share many biochemical properties with *Vibrio* species, with which they were jointly classified in the family *Vibrionaceae* until genotypic information provided new insights. *Aeromonas* species are increasingly associated with gastrointestinal infections, albeit with great apparent variation in pathogenicity and virulence both between and within species of the genus. We report two cases with clinically mild cholera-like symptoms, at a time when a cholera outbreak was unfolding in other regions of the country (Tanzania). These are the first cases to be reported with *Aeromonas* mimicking cholera in our area.

**Case presentation:**

Two patients were admitted at the isolation unit designated by the Kilimanjaro Christian Medical Centre for emerging infectious diseases and provided informed consent about regular stool analysis and culture under the provisional diagnosis of gastroenteritis. The first patient was a 23-year-old black African woman with a 2-day history of watery diarrhea and vomiting associated with a temperature of 39.7 °C. The second patient was a 47-year-old black African woman with a 2-day history of diarrhea and vomiting with a temperature of 37.7 °C, and she was hemodynamically stable. Both patients were isolated in a specific area for infection control and treated with fluids and orally administered rehydration solution, ciprofloxacin, metronidazole, and paracetamol. Stool culture was done. The isolated colonies were reported as *V. cholerae* and transferred to the research laboratory of Kilimanjaro Clinical Research Institute for confirmation using whole genome sequencing. Microbiological testing determined colonies isolated from stool to be *V. cholerae*, and warranted the conclusion “presumptive cholera.” Whole genome sequencing, however, established the presence of *Aeromonas caviae* rather than *V. cholerae*.

**Conclusions:**

The co-existence of *Aeromonas* species with *V. cholerae* in cholera-endemic regions suggests the possibility that a proportion of suspected cholera cases may be *Aeromonas* infections. However, with close to no epidemiological data available on *Aeromonas* infection in cases of diarrhea and dysentery in Sub-Saharan Africa, it is not currently possible to establish the extent of misdiagnosis to any degree of certainty. Whole genome sequencing was shown to readily exclude *V. cholerae* as the etiological agent and establish the presence of *Aeromonas* species*.*

## Background

Detection of *Aeromonas* species, and in particular differentiating these from *Vibrio* species, can require a large number of laboratory tests [1–3]. We were caught out by this when on the same day two patients presented with cholera-like symptoms at a time when a major cholera outbreak was unfolding in several other regions in the country (Tanzania). We present this case report as a cautionary tale, with possible relevance for the management of cholera outbreaks. Having obtained the whole genome sequences of the isolates we also hope to make a modest contribution to the growing evidence linking *Aeromonas* with human enteropathy.

Tanzania experienced two large cholera outbreaks in 2015. In a refugee camp on the Tanzania–Burundi border, 4833 cases had been reported, of which 40 died [4]. A new index case presented in Dar es Salaam on 15 August 2015. Our cases presented on 8 September 2015, when 857 cases and 13 deaths had been reported countrywide. By October, the outbreak had spread to 13 regions, with 4835 cumulative cases [5, 6]. When the outbreak abated in April 2016, a total of 24,018 cases had been reported in 28 regions, of which 429 died [7].

Cholera is an acute diarrheal infection caused by ingestion of food or water contaminated with the bacterium *Vibrio cholerae*. Cholera is a potentially fatal disease affecting adults and children, and can kill within hours if untreated. The O1 and O139 serogroups of *V. cholerae* cause outbreaks; other strains can cause mild diarrhea but do not generate epidemics. Approximately 80% of people infected with *V. cholerae* do not develop symptoms; they can, however, shed the bacteria for up to 10 days. Among those who develop symptoms, 80% have mild or moderate symptoms, while around 20% develop acute watery diarrhea with severe dehydration [8].

*Aeromonas* species have been associated with a wide variety of human infectious diseases including gastroenteritis, wound infections, septicemia, respiratory infections, hepatobiliary infections, and urinary tract infections [3, 9]. *Aeromonas* is most commonly associated with gastrointestinal infections. Most *Aeromonas*-associated enteropathy is watery diarrhea with a self-limited course; however, fever, abdominal pain, vomiting, bloody diarrhea, and secondary dehydration may occur. Outbreaks of *Aeromonas*-associated enteropathy have rarely been reported. In one outbreak of diarrhea with 2170 cases, *Aeromonas* was the most prevalently isolated single enteric pathogen (82%), but *V. cholerae* strain O1 was also isolated in 12% of samples, and identified as the more likely etiological agent [10].

*Aeromonas* is ubiquitous in aquatic environments. *Vibrio* and *Aeromonas* have been shown to co-inhabit a shared host. The non-biting midges (chironomids), an abundant insect in aquatic habitats [11], have been proposed as a natural reservoir for *Vibrio* and *Aeromonas* species [12].

Very little is known about the incidence of *Aeromonas*-associated gastroenteritis in Sub-Saharan Africa (SSA). Studies in a number of Asian, Latin-American, and African countries isolated *Aeromonas* in 1 to 80% of children with diarrhea, and in 0 to 45% of asymptomatic children [13]. The recent large-scale Global Enteric Multicenter Study (GEMS) study [14] found *Aeromonas* infection to be significantly associated with watery diarrhea and dysentery in children under 5 in Bangladesh and Pakistan, but found no significant attributable risk for the sites in India or the four SSA countries in the study. In Bangladesh and Pakistan, the study found *Aeromonas* in 18.7 to 28.6% of stools of children with medium to severe diarrhea, usually with co-infecting enteropathogens. It was found as the sole pathogen in only 1.8 to 4.2% of cases. *Aeromonas* was detected in the stools of approximately equal numbers of cases and matched controls [14–16].

The epidemiology of *Aeromonas*-associated diarrhea is influenced by host and environmental factors, and by variation in pathogenicity between strains [17, 18]. Isolates were shown to exhibit large variation in the presence of virulence genes, both between and within species [17, 19, 20].

Up to the 1980s, the genera *Aeromonas* and *Vibrio* were classified jointly in the family *Vibrionaceae.* Molecular genetic developments then entailed reclassification into its proper family of *Aeromonadaceae*. Several reclassifications within the genus have since taken place. At least 31 acknowledged or proposed species of *Aeromonas* have been described, exhibiting great phenetic diversity [17]. The dominant species associated with human enteropathology are *Aeromonas caviae*, *Aeromonas hydrophilae*, and *Aeromonas veronii* biovar sobria, although studies disagree on the relative importance of these species in human infections [13, 21–24].

To distinguish *Aeromonas* species from their former family members *V. cholerae* and *Plesiomonas shigelloides* (all oxidase positive, setting them apart from the *Enterobacteriaceae*), the key screening reactions include: growth in nutrient broth in the presence and absence of salt; growth on thiosulfate-citrate-bile salts-sucrose (TCBS) agar (selective for *V. cholerae* with circular yellow or green > 2 mm colonies, inhibitive to *Aeromonas* with no or tiny colonies); resistance to vibriostatic agent O/129; and the string test [3]. Ghenghesh *et al*. (2008) documented laboratory procedures requiring minimal resources, enabling application in low-resource settings [13]. *Aeromonas* strains have been documented to exhibit atypical results for various assays and can in rare cases “be mistakenly identified as *V. cholerae* if a complete battery of screening tests aren’t performed” [1]. The UK’s Public Health England standard for identification of *Vibrio* species was expanded in 2015 to include procedures for differentiation of *Aeromonas* species [25].

Genomic data will readily and accurately identify isolates as members of the *Aeromonas* genus, although typing to species rank is complicated by multiple factors. Classification by comparison with publicly deposited genomes, even in curated databases, can result in mislabeling [22]. This is in part due to posterior reassignments within the genus, but also inherent to *Aeromonas* biology. Recombination occurs frequently between members of the genus, creating “highways of gene transfer” that obscure vertical phylogeny [26]. In addition, the ribosomal RNA (rRNA) genes of *Aeromonas* species are characterized by an exceptionally high copy number and large intragenomic heterogeneity, defying the common identification measure of 16S rRNA gene identity [27–29].

In light of the intricate taxonomy of *Aeromonas*, whole genome content comparisons, as measured by *in silico* DNA-DNA hybridization and average nucleotide identities (ANI), have been proposed as the “new gold standard” for identification [22, 26]. *In silico* multi-locus sequence typing (MLST) using the conserved housekeeping genes *gyrB*, *rpoD*, *recA*, *dnaJ*, *gyrA*, and *dnaX* was demonstrated to accurately reproduce the taxonomy of *Aeromonas* species [30].

For clinical purposes, detailed identification is arguably less relevant than determining the virulence and drug susceptibility profile of the organism, in particular when these characteristics do not segregate cleanly with species. Whole genome sequencing (WGS) has the benefit of providing data to analyze both, to the extent that mechanisms have been genotyped. WGS can additionally provide unprecedented insight into the dynamics of disease outbreaks.

With outbreaks recurring almost every year, cholera is an endemic disease in Tanzania, as in many other SSA countries. Its etiological agent, *V. cholerae*, co-exists with species of the phenotypically similar but genotypically distinct genus *Aeromonas* in the same region. Clinical cases of mild to severe infectious gastroenteritis may be caused by virulent strains of *Aeromonas*, although very little is known about its incidence in SSA. Laboratory tests for *V. cholerae* may give false positive results when assayed on *Aeromonas* species*.* Therefore, advanced testing – which is often unavailable in low-resource settings – can be required to establish *Aeromonas* infection, as this case report intends to illustrate.

## Case presentation

Two patients were admitted at the isolation unit designated by the hospital for an emerging infectious diseases outbreak in one of the referral hospitals in Moshi, Tanzania, which is a tertiary referral hospital and academic training center. The two patients provided informed consent about regular stool testing with the indication of infectious gastroenteritis, as well as for analysis of the samples in further genomic studies.

The first patient was a 23-year-old black African woman with a 2-day history of diarrhea and vomiting. The day prior to admission, she had eaten a fried meat snack from a restaurant. Later in the night she had more than ten bouts of profuse yellowish-greenish and watery diarrhea, with colicky abdominal pains, vomiting only once. On examination, she was sick-looking but alert, not dehydrated, hemodynamically stable, and had 39.7 °C fever.

The second patient was a 47-year-old black African woman with a 2-day history of diarrhea and vomiting. She came straight from her home, and had eaten her normal meal of meat, ugali (a starch provider), and vegetables, preparing the food as she always did. She had over ten bouts of profuse watery diarrhea, greenish-yellowish in color and associated with generalized abdominal pains that were colicky in nature, and vomited five times. On examination, she was sick-looking, alert, and not dehydrated with a temperature of 37.7 °C, and she was hemodynamically stable. Both patients were isolated in a specific area for infection control and treated with fluids and orally administered rehydration solution, ciprofloxacin, metronidazole, and paracetamol.

### Microbiology

In both patients, stool analysis and culture were done under the provisional diagnosis of gastroenteritis. The stool samples from the two patients at the Kilimanjaro Christian Medical Centre (KCMC) were subjected to a number of standard assays routinely performed in the case of suspected infectious gastroenteritis at the KCMC Clinical Laboratory. Stool microscopy assessment was done on Gram staining and wet preparation for motility. Rectal swabs were put in alkaline peptone water (APW; Sigma; Steinheim, Germany) for enrichment and incubated at 37 °C for 18 hours. The enriched samples were then subcultured onto TCBS agar (Sharlau, Spain). After 24 hours of incubation at 37 °C, yellow colonies (sucrose fermenting, 2–3 mm diameter) suspected as *Vibrio* cholera were characterized by Gram staining (Gram-negative comma-shaped rods), and biochemical testing was done for positive oxidase reaction, urea hydrolysis, citrate fermentation, sulfur reduction, and indole production in sulfide indole motility (SIM) medium. Serological typing by agglutination test with the polyvalent O1 antiserum was done on one isolate from case 2. Inaba and Ogawa sero-agglutination was not performed. Results are summarized in Table 1.

**Table 1:** Microbiology results

Test:	KIA	Urea	Citrate	SIM	Gas	Oxidase	Poly O1	Inaba / Ogawa
Case 1	Acid / Acid	Neg	Neg	Neg / Neg / Pos	Neg	Pos	ND	ND
Case 2	Acid / Acid	Neg	Neg	Neg / Neg / Pos	Neg	Pos	Pos	ND

*KIA* Kligler Iron Agar, *ND* not done, *Neg* negative, *Pos* positive, *SIM* sulfite indole motility

The isolated colonies were reported as presumptive *V. cholerae*, but the inconclusive evidence and discordance with clinical presentation necessitated confirmatory tests. Medication was changed to doxycycline for another 3 days. The patients progressed well, and stool samples were taken again for follow-up analysis. A full blood picture was essentially normal in both patients.

### Genomics

The isolates were transferred to the research laboratory of Kilimanjaro Clinical Research Institute (KCRI) for confirmation using WGS. KCRI is the research institute liaised to the referral facility and hosts one of the very few next generation sequencers in East Africa. Here the isolates were subcultured onto TCBS agar plates and incubated at 37 °C for 24 hours. Colonies from TCBS were used for Genomic DNA extraction. Genomic DNA was extracted using MasterPure™ Complete DNA and RNA Purification Kit (Epicentre, Illumina Inc.). The quality and quantity of genomic DNA were accessed using Qubit 2.0 fluorometer (Invitrogen, Life Technologies). DNA library preparation (dual indexing) was done using Nextera XT DNA preparation kit (Illumina Inc.). WGS of the library was done on Illumina MiSeq (Illumina Inc., San Diego, USA) using 2 by 250 bp paired end protocol.

Initial genomic analysis of the sequencer reads was performed using the standard pipeline for WGS research at KCRI. The pipeline is built from local instances of the free online services at the Centre for Genomic Epidemiology of Danish Technical University (https://cge.cbs.dtu.dk/services/). The pipeline successively performed: identification of species from raw reads (KmerFinder 1.1); *de novo* genome assembly and quality assessment (SPAdes 3.5, Quast 2.3); 16S rRNA species typing (SpeciesFinder 1.2); multi-locus typing (MLST 1.8); and antimicrobial resistance prediction (ResFinder 1.3). Additional analyses were performed to disambiguate the pipeline output, and investigate virulence genes (BLAST 2.2).

The sequencer run produced a below average yield (average base coverage 10.4), and as a consequence *de novo* assembly resulted in fragmented genome assemblies. Both samples produced approximately 450 contigs, with largest contig ~ 90 kbp, and N50 size ~ 20 kbp. This affected the discriminatory power of downstream analyses and precluded an exhaustive phylogenetic analysis, but sufficed for the clinical purpose of species identification.

KmerFinder results conclusively excluded *V. cholerae*, and established the isolates as genus *Aeromonas*, most likely species *A. hydrophila*. SpeciesFinder failed to 16S-type the assemblies, probably as a consequence of *Aeromonas*’ characteristically high 16S rRNA gene copy number, exacerbated by the problems that long repeats pose for genome assembly. Typing was then performed against the hand-curated GreenGenes 16S and SILVA SSU/LSU reference datasets. Neither provided a taxonomic assignment more specific than genus *Aeromonas*. Subsequent local alignment of the assemblies to the NCBI 16SMicrobial database, and manually collating the matching contigs, produced sequences which for both isolates matched *A. caviae* (GenBank sequence NR_104824.1).

Conclusive evidence for species *A. caviae* was obtained from matching the six housekeeping genes identified by Martinez-Murcia *et al*. [30] against reference sequences. Both isolates had 100% identity matches with *A. caviae* strains on the *gyrB* and *recA* genes, > 99.6% on the *rpoD* and *gyrA* genes, and > 98.5% on the *dnaJ* and *dnaX* genes. The two KCMC isolates matched different alleles for multiple genes, proving it unlikely that the infections originated from a single source.

ResFinder detected the *blaMOX-4* (> 95.2% identity) and *ampH* genes (> 82.4% identity) coding for beta-lactam resistance in both isolates. In one isolate it detected the *sul1* and *dfrA-15* genes with 100% identity, coding for sulfonamide and trimethoprim resistance respectively.

The fragmented assembly prevented an exhaustive search for proposed virulence genes. The *alt* gene for thermo-labile cytotonic enterotoxin was found integrally in both isolates. The *act* gene for cytotoxic enterotoxin was absent in both. For several other genes (*ast*, *hlyA*, *aerA*, *aexT*, *ascV*) matching partial fragments (350–700 bp) were found, providing insufficient support to establish their presence.

## Discussion

We report concomitant outbreaks of genuine *V. cholerae* infection along with cases of a less infectious and less virulent clinical cholera-mimic caused by *A. caviae* in the United Republic of Tanzania.

The initial microbiological tests which showed positivity for *V. cholerae* were an obvious reason for concern. An outbreak of cholera type O1 – or in our case the spread of an ongoing outbreak to a new and populous region – has widespread consequences. Routine further testing by the clinical laboratory did not disprove the hypothesis that the pathogen was *V. cholerae*. The suspicion of an alternative explanation for the gastroenteritis was raised by the clinically mild phenotype in the two patients from our hospital. This underscores the importance of clinical and laboratory scrutiny, especially when typical cases have been reported from the same greater region.

*Aeromonas* species may yield false positive microbiological test results for *V. cholerae*. The extent to which misdiagnosis occurs is unknown. Provisionally confirmed cases in suspected outbreaks which are rapidly contained probably go unreported. Not all O1 infections are fulminant, and all non-O1 *V. cholerae* infections have a clinical presentation which is very similar to that of *Aeromonas* enteric infection. The fact that in a separate study (in progress) we isolated *Aeromonas* from 2 out of 15 stool samples from cases of the 2015 cholera outbreaks, suggests that there is some extent of co-occurrence, and possible confounding, of the two infections during O1 outbreaks.

WGS straightforwardly produced the data required for species and strain identification, at a comparable cost to the USD 85–90 for sero-agglutination tests [31]. However a number of conditions must be met before WGS can be used as the “new gold standard.” Curated reference sequences with validated taxonomical assignment must be available, and there must be agreement on decision procedures for establishing species identity. The case of *A. caviae* also demonstrates that automated bioinformatics pipelines which do not take into account species-specific genotypic idiosyncrasies may fail to correctly identify species. Some *Aeromonas* species defy 16S rRNA typing even under the assumed ideal conditions of WGS, and need bespoke analysis. More generally, in order that reliable conclusions can be drawn from the diagnostics they provide, bioinformatics instruments need validation and calibration in terms of sensitivity and specificity. This gap particularly needs closing when WGS moves from research-only to clinical applications.

### Strength and limitation

The strength of our cases was the isolation of *Aeromonas* species supported by availability of the WGS at KCRI and avoidance of a misdiagnosis. A limitation might be that the cases were from an isolation unit where cholera-suspected cases were admitted; as they were the only two cases admitted to the unit during the cholera outbreak there might be a possibility of missing other cases of gastroenteritis due to *Aeromonas* species in other wards in the hospital.

## Conclusions

This case report shows that suspected cases of *V. cholerae* infection may require extensive testing to exclude *Aeromonas* species, notably *A. caviae*. It highlights the need for laboratory guidelines such as the UK guidelines [25] which pay attention to the possible confusion, ideally adapted to low-resource settings, to be applied in suspected cases of *V. cholerae*. WGS was shown to readily exclude *V. cholerae* as the etiological agent and establish the presence of *Aeromonas* species. WGS however is not routinely available, certainly not in Africa, nor will it soon replace classical microbiology as the first-line diagnostic for cases of enteropathy.

The co-occurrence of *V. cholerae* and *Aeromonas* species in cholera-endemic regions suggests the possibility that a proportion of suspected cholera cases may instead exhibit *Aeromonas*-associated gastroenteritis. However, the dearth of epidemiological data on *Aeromonas* infection in cases of diarrhea and dysentery, and in particular its near complete lack for SSA, makes it impossible to establish the extent of misdiagnosis with any degree of certainty.

However, if the qualification “*an emergent human pathogen*,” which is almost idiomatically used in the literature on *Aeromonas* species, is ever to be promoted to “*an endemic human pathogen*,” then there is a strong need for both comprehensive epidemiological data and reliable means of diagnosis.

## Patient’s perspective

On discussion of the result with one of the patients and the publication plan, she said that it was good that she knew she had not had cholera because she had had feelings of shame and stigma. She said: “I was wondering as how can I alone get cholera while the same food I prepared myself my children ate but none of them had similar disease as me, now I am happy and will tell the family I had no cholera.”
